# Novel Graphene Oxide Nanohybrid Doped Methacrylic Acid Hydrogels for Enhanced Swelling Capability and Cationic Adsorbability

**DOI:** 10.3390/polym13071112

**Published:** 2021-04-01

**Authors:** Yufei Liu, Ying Lyu, Yongqin Hu, Jia An, Rubing Chen, Meizhu Chen, Jihe Du, Chen Hou

**Affiliations:** 1 Key Laboratory of Optoelectronic Technology & Systems, Chongqing University, Ministry of Education, Chongqing 400044, China; yufei.liu@cqu.edu.cn (Y.L.); lyuying@cqu.edu.cn (Y.L.); Huyongqin@cqu.edu.cn (Y.H.); anjia@cqu.edu.cn (J.A.); chenrubing@cqu.edu.cn (R.C.); cmz@cqu.edu.cn (M.C.); dujihe@cqu.edu.cn (J.D.); 2 Centre for Intelligent Sensing Technology, College of Optoelectronic Engineering, Chongqing University, Chongqing 400044, China; 3 Centre for Nano Health, College of Science, Swansea University, Singleton Park, Swansea SA2 8PP, UK

**Keywords:** nanohybrid hydrogel, ion and pH response, cationic dyes adsorbent

## Abstract

Novel versatile hydrogels were designed and composited based on covalent bond and noncovalent bond self-assembly of poly(methacrylic acid) (PMAA) networks and nanohybrids doped with graphene oxide (GO). The structures and properties of the neat PMAA and the prepared PMAA/GO hydrogels were characterized and analyzed in detail, using X-ray diffraction (XRD), scanning electron microscopy (SEM), Fourier transform infrared (FTIR) spectroscopy, swelling and cationic absorption, etc. The swelling results showed that the water penetration follows the non-Fick transport mechanism based on swelling kinetics and diffusion theory. The swelling capacity of PMAA and composited PMAA/GO hydrogels toward pH, Na^+^, Ga^2+^, and Fe^3+^ was investigated; the swelling ratio was tunable between 4.44 and 36.44. Taking methylene blue as an example, the adsorption capacity of PMAA/GO hydrogels was studied. Nanohybrid doped GO not only self-associated with PMAA via noncovalent bonding interactions and had a tunable swelling ratio, but also interacted with water molecules via electrostatic repulsion, offering a pH response of both the network and dye absorption. Increases in pH caused a rise in equilibrium swelling ratios and reduced the cumulative cationic dye removal.

## 1. Introduction

Hydrogels, either chemically or physically crosslinked, are mostly spontaneously formed as they capture water within their structure, having excellent and unique characteristics such as high hydrophilicity, elastic structure, and smart stimulus response, suitable for biomedicine [1,2], artificial muscles [3], robot actuators [4,5], adsorbents of toxic chemicals and wastewater [6,7], and drug delivery [8,9,10,11] applications. Their characteristics are dependent on changes in the external environment, such as pH, ionic strength, electrical stimulation [12], and temperature. The advantages of physical hydrogels over chemical hydrogels are that they form a crosslinked structure by associative forces capable of forming noncovalent crosslinks, and do not require any crosslinking agents. With physical crosslinking, the polymer chains are held by entanglements or by some physical interactions, such as hydrogen bonding [13,14] and ionic interactions [15,16,17]. However, industrial waters and natural waters are often contaminated by toxic or carcinogenic impurities, causing ecological disequilibrium and severe public health problems [18]. Despite the considerable advances in the field of water treatment [19,20], until now, less research has been conducted on the elimination of organic pollutants such as cationic dyes. Recently, attention has been paid to producing pollutant adsorbents from acrylic polymers [21] such as methacrylic acid due to their advantages: abundance, mild reaction conditions, and being biodegradable in nature [22]. Wu et al. synthesized vitamin C (VC)-modified reduced graphene oxide hydrogel (V-RGOH) using a green and facile self-assembly process with the assistance of biocompatible VC molecules for high-performance NH_3_ and NO_2_ [23]. Alammar et al. engineered nanocomposite hydrogels based on sustainable cellulose acetate for water treatment [24]. Cao et al. applied the open framework structure Na_3_V_2_(PO_4_)_3_@C as a novel faradaic electrode in the hybrid capacitive deionization (HCDI) system, and its outstanding performance demonstrated its promise as a material for desalination [25].

Graphene oxide (GO), which is prepared by chemical oxidation of natural graphite, is a precious nanomaterial used for many applications, such as for medical [26], energy storage [27], flexible sensor [28], and wastewater treatment [29] purposes, due to the abundant oxidic groups on its surface and its excellent specific surface area [30]. GO can be evenly dispersed into an aqueous phase to form strong interactions with polymers [14,31], which can accelerate the gelation of organic pollutant solutions. Sarkar et al. synthesized a new graphene oxide-based two-dimensional (2D) crosslinker (GOBC), and exploited the functionality of this crosslinker for the enhancement of toughness and stretchability of a poly(acrylic acid) (PAA) hydrogel [32]. This hydrogel will be used for cutting-edge applications in biomedical engineering. Ren et al. reported the facile preparation of a 3D regenerated cellulose/graphene oxide composite [33]. Alammar et al. developed polybenzimidazole (PBI), graphene oxide (GO), and reduced GO (rGO) nanocomposite membranes by the common blade coating and phase inversion technique, which can be used for the treatment of water produced by the oil and gas industry. This nanocomposite membrane exhibits the expected long-term performance (up to 180 days), and excellent reusability [34]. Zhang et al. developed a simple and green in situ assembly and reduction approach that can be used for the fabrication of reduced graphene oxide-supported palladium nanoparticles (Pd/rGO). The material was reported to have excellent catalytic performance and reusability (at least six times) [35]. Mikhaylov et al. proposed a green synthesis method, combined with graphene oxide reduced by tin disulfide, to obtain a highly stable sodium-ion battery anode [36].

In this context, the aim of our study was to prepare a cationic dyes absorbent. We carefully constructed a synthetic scheme, and a novel nanohybrid hydrogel was designed and developed in which a self-assembly approach was introduced by the hydrogen-bonding association of GO with covalently linked PMAA hydrogels. We discussed the swelling capacity of PMAA and PMAA/GO hydrogels toward pH, Na^+^, Ga^2+^, and Fe^3+^. The adsorption capacity of PMAA/GO hydrogels to methylene blue dye was investigated. The removal rate was as high as 96.92%. Increasing pH values resulted in increasing equilibrium swelling ratios, and in cumulative removal being reduced from 93.24% to 77.85%.

## 2. Experiments

### 2.1. Materials

High-purity scaly graphite (300 mesh, 99.6%) was purchased from XFNANO Corp. (Jiangsu, China). Methacrylic acid (MAA, analytical reagent, 98%), potassium persulfate (KPS, analytical reagent, ≥99%), and methylene blue (MB) were purchased from Aladdin Chemical Reagent Corp. (Shanghai, China). Potassium permanganate (analytical reagent, ≥99.5%), sodium hydroxide (analytical reagent, >99%), and 30 wt% hydrogen peroxide (H_2_O_2_) were purchased from Chongqing Chuandong Chemical Reagent Corp. Concentrated sulfuric acid (98%), hydrochloric acid (36–38%), phosphorus pentoxide (P_2_O_5_, analytical reagent, 98%), and N,N′-methylene-bis(acrylamide) (MBA, analytical reagent, 98%) were purchased from Chengdu Kelong Chemical Reagent Factory. All experimental water was deionized water (18.2 MΩ). All reagents were used as purchased.

### 2.2. Methods

#### 2.2.1. Synthesis of Graphene Oxide (GO)

The modified Hummers method [37,38] was used to prepare GO. First, 1 g high-purity scaly graphite with 30 mL concentrated sulfuric acid, 0.8 g KPS, and 0.8 g P_2_O_5_ were mixed and reacted at 80 °C for 4 h and stirred in a round-bottom flask. After the reaction mixture was cooled to room temperature, it was diluted with 1 L ultrapure water to obtain a black mixture, and then the mixture was left to stand overnight. We filtered the overnight mixed solution, using the ultrapure water to remove the residual acid, and then dried the reactant overnight at 60 °C. The product was dispersed in 40 mL concentrated sulfuric acid at 0 °C again, and 5 g KMnO_4_ was slowly added to the mixture under an ice bath, mixed, and stirred for 2 h at 35 °C. The final mixed solution turned grass green. We slowly added 83.4 mL ultrapure water to dilute the mixed solution, continued to stir the solution for 2 h, and then added 234 mL ultrapure water and 6.7 mL 30 wt% hydrogen peroxide slowly and sequentially. The color of the mixed solution turned bright yellow. The mixed solution was dialyzed to neutral, then centrifuged and freeze-dried to obtain GO, which was stored in a dry environment until use.

#### 2.2.2. Preparation of PMAA/GO Hydrogel

In situ synthesis was used to prepare the PMAA/GO hydrogel. We weighed MAA to obtain a 3 mol/L aqueous solution, and a certain amount of GO was dissolved in ultrapure water to obtain a 4 mg/mL GO dispersion, which was then mixed with 2 mL MAA (3 mol/L), 2 mL ultrapure water, and MBA uniformly, in which the MBA/MAA molar ratios were 0.25, 0.5, 1, 2, 3, and 4 mol%. Then, we added the initiator KPS, in which the KPS/(MAA + MBAM) molar ratios were 0.5, 1, 2, 3, and 4 mol%. Finally, the solution was reacted at 70 °C for 12 h. For the synthesis of GO composite hydrogel, we ensured that the molar ratio of KPS/(MBAM + MAA) was 1 mol%, and the other amounts remained unchanged. We replaced the ultrapure water with 2 mL of 4 mg/mL GO dispersion.

#### 2.2.3. Structural Characterization

In this study, the hydrogel was freeze-dried and fractured with liquid nitrogen, and then the fractured surface was sprayed with gold to obtain an SEM sample. Next, an EVO18 analytical scanning electron microscope (SEM, Zeiss, Germany) was used to characterize the appearance of the hydrogel. We used a UV-2700 UV–vis spectrophotometer (Shimadzu, Japan) to determine the absorbance of the MB solution, a Nicolet IS10 infrared spectrometer to analyze the components of the sample, and a BRUKER D8 ADVANCE X-ray powder diffractometer to analyze whether the composite hydrogel was successfully composited.

#### 2.2.4. Swelling Rate Characteristic of PMAA/GO Hydrogel

The hydrogel samples were naturally dried at room temperature. Then, the samples were weighed and immersed in deionized water under ambient conditions. Afterward, the sample was removed at regular intervals, the surface water was wiped off with filter paper, and the sample was weighed. We estimated the swelling rate of the sample according to [39]:

(1) $$W_{A}=\frac{\left(W_{t}-W_{d}\right)}{W_{d}}$$

where *W_t_* is the weight of the samples after swelling at a certain point and *W_d_* is the weight of the dried sample. The *W_A_* values of samples in the NaCl, CaCl_2_, and FeCl_3_ saline solutions (1 mM) were analyzed. At room temperature, the swelling rates at different pH values (3.0, 5.0, 7.0, and 9.0) were tested.

#### 2.2.5. Adsorption Performance of Hydrogels for Methylene Blue

A certain amount of dry PMAA/GO hydrogel was added to 0.1 mg/mL methylene blue (MB) solution (selecting 0.1 mg/mL as the original concentration was convenient and ensured the accuracy of tests with the UV-2700). Then, the solution was placed in a constant-temperature water bath shaker for 2 h to reach adsorption equilibrium. We used an ultraviolet spectrophotometer to test the UV–vis spectra before and after the adsorption of the MB solution. We substituted the corresponding absorbance into the standard curve of the MB solution to obtain the concentration before and after the adsorption of the MB solution. According to Equations (2) and (3), we calculated the adsorption amount and dye removal rate [29,40]:

(2) $$Q_{e}=\frac{\left(c_{0}-c_{e}\right)v}{m}$$

(3) $$\mathrm{Dye}\;\mathrm{removal}\;(\%)=\left(c_{0}-c_{e}\right)/c_{0}\times 100\%$$

where *Q_e_* is the adsorption amount at adsorption equilibrium, *c*_0_ is the concentration before adsorption of MB solution, *c_e_* is the concentration at adsorption equilibrium of the MB solution, *ν* is the volume of the solution, and *m* is the mass of the adsorbent.

## 3. Results and Discussion

**Figure 1:**
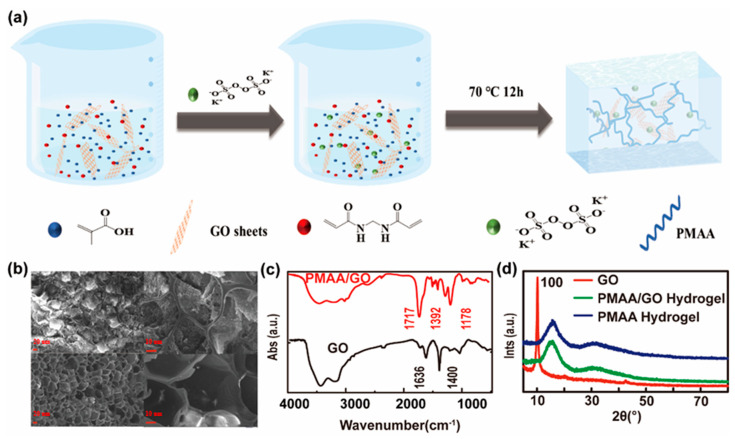
Scheme of synthesis and characterization of poly(methacrylic acid) (PMAA)/graphene oxide (GO) hydrogel. (**a**) Synthesis scheme of PMAA/GO hydrogel. (**b**) SEM images of PMAA and PMAA/GO nanohybrid hydrogel. (**c**) FTIR of PMAA/GO nanohybrid hydrogel and GO. (**d**) XRD patterns of GO, PMAA hydrogel, and PMAA/GO composite hydrogel. Scale bars are 10 μm and 20 μm.

The preparation scheme of the PMAA/GO hydrogel is shown in Figure 1a. A simple in situ synthesis method was used to prepare the PMAA/GO hydrogels. GO was the filler, which was dispersed in the mixture solution. The SEM images of the intersection surface of the freeze-dried hydrogel samples of the prepared PMAA and PMAA/GO (GO/MAA = 4.8 wt%) hydrogels are shown in Figure 1b. The cross-sections of the samples exhibited a micro-porous structure. The images of the bottom of the PMAA/GO hydrogel showed bigger and more ordered pores than the top of the PMAA. An FTIR spectrophotometer was used to determine the functional groups of the original GO and the prepared PMAA/GO hydrogel. The spectra were recorded from 4000 to 400 cm^−1^. Figure 1c shows the FTIR spectra of the original GO and the prepared PMAA/GO. In Figure 1c, for GO, a broad absorption band at 3443 cm^−1^ is shown due to the stretching frequency of the –OH group on the surface of GO. The presence of a sharp absorption band at 1636 cm^−1^ confirmed the presence of a –C=O group. The strong bands around 1400 cm^−1^ are assigned to –CH_2_ bending vibration. The result showed that GO was successfully GO. In Figure 1c, for the PMAA/GO hydrogel, the presence of a broad absorption band at 3454 cm^−1^ is due to the overlap between the O–H stretching and N–H stretching vibrations of the NH group. The sharp band at 1717 cm^−1^ is due to the carbonyl group (C=O stretching) of the amide group of MAA, and the band at 1392 cm^−1^ is due to the bending of the NH_2_ group; the presence of an absorption band at 1178 cm^−1^ is due to a C–C bond. These bands characterize both GO and PMAA bonds. The previous peaks verified the formation of the PMAA/GO hydrogel. Figure 1d presents the XRD patterns of the original GO, prepared PMAA/GO, and PMAA hydrogels. The PMAA hydrogel showed a relatively significant diffraction peak at 2θ = 19.74° and a broad and weak peak near 2θ = 30.7°, which indicated that there was a certain amount of ordered crystal configuration in the PMAA hydrogel. For the PMAA/GO-doped hydrogels, the characteristic diffraction peaks of PMAA were only shown at 2θ = 19.74° and 2θ = 30.7°, which indicated that the crystal structure of the PMAA hydrogel was not affected by GO incorporation. However, the characteristic diffraction peak of GO (2θ = 9.92°) disappeared in the XRD spectrum of the PMAA/GO hydrogel, which indicated that the GO flakes were exfoliated and dispersed well, that it is well-compatible with the PMAA polymer network, and that the PMAA polymers are entangled with each other to form a PMAA/GO composite hydrogel.

**Figure 2:**
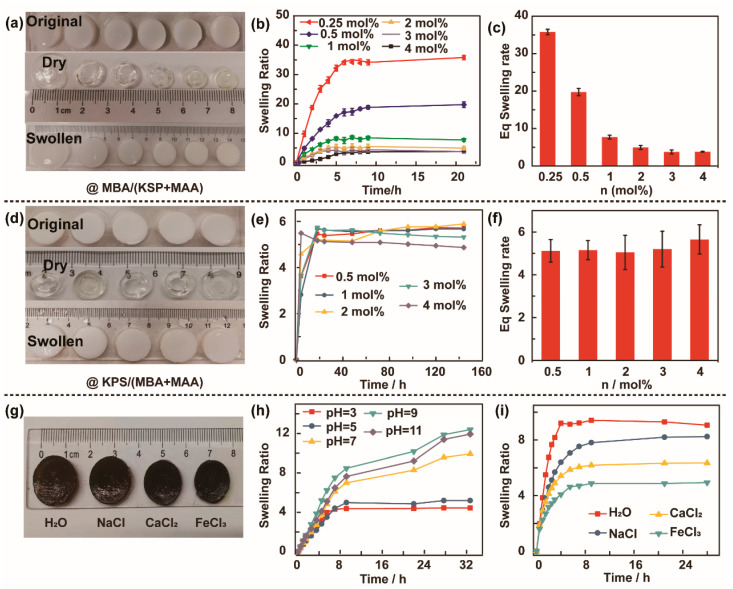
Swelling properties of PMAA hydrogel and PMAA/GO composite hydrogel. (**a**–**c**) Determination of gel swelling rate with different molar ratios of N,N′-methylene-bis(acrylamide) (MBA)/MAA. (**a**) Comparison before and after swelling; (**b**) trend in swelling rate over time; (**c**) the swelling rate corresponding to the swelling equilibrium. (**d**) Comparison before and after swelling; (**e**) trend in swelling rate over time; (**f**) the swelling rate corresponding to the swelling equilibrium. (**g**) Photos of PMAA/GO gels at swelling equilibrium under different salt solutions. (**h**) Influence of pH on swelling rate of PMAA/GO gel. (**i**) The variation in PMAA/GO gel swelling rate with time in different salt solutions.

Swelling is an important factor for the applicability of chemical crosslinking hydrogels. The crosslinking degree can immediately result in a dramatic change in the hydrogel’s swelling behavior. Figure 2 shows the effect of crosslinking and the initiating agent’s concentration on the swelling ratio of the prepared series of the PMAA (MBA%) and PMAA (KPS%) hydrogels. Figure 2a shows the PMAA (MBA%) hydrogels in different states: the white original, the transparent dry, and white swollen hydrogels from top to bottom. The equilibrium swelling behavior of the PMAA (MBA%) hydrogels was calculated using Equation (1) as a function of time at different crosslinking agent concentrations, as presented in Figure 2b. We found that the content of MBA was significantly affected by the swelling ratio. The swelling behavior was lowest at MBA 4 mol%; as MBA content increased in the hydrogel composition, the swelling behavior decreased. The swelling ratio was only about 3.75 after 5 h. This may be due to the increase in the crosslinking density, which resulted in narrowing of the pore size and reduced the free spaces available for water retention, leading to a decrease in the osmotic force driving the water into the hydrogel network. As a result, the equilibrium (Eq) swelling ratio decreased, as displayed in Figure 2c. The Eq swelling ratio decreased from 36.19 to 3.75, whereas the swelling behavior of the PMAA (KPS%) hydrogels did not show significant changes with increasing KPS (Figure 2d). No remarkable morphology change occurred when immersed in water. The water absorption of the PMAA (KPS%) hydrogel decreased with increasing KPS content, from 0.5 to 4 mol% in the polymer network (Figure 2e), as expected. KPS is a radical initiator. Incorporation of PMAA in the crosslinked network decreased the hydrophilicity of the network, which ultimately led to the reduction in water absorption of the hydrogel. The Eq swelling ratio is shown in Figure 2f. High crosslink density not only plays a role in reducing the water absorption of the hydrogel, but it also makes it brittle. Graphene oxide (GO) nanolayers were introduced as a filler to the PMAA/GO hydrogel. The hybrid PMAA/GO hydrogel was sensitive to pH and metal ions with different valence states. The swelling performance of immersion in different salt solutions, such as NaCl, GaCl_2_, and FeCl_3_, is displayed in Figure 2g. The water absorption of the PMAA/GO hydrogel decreased with the increase in valence state. The water absorption is increased with an increase in pH, as shown in Figure 2h; that is, the swelling ratio increased from 4.44 at pH = 3 to 12.4 at pH = 9 when immersed after 32.7 h, because increasing pH is supposed to increase the water absorption of hydrogels. During the synthesis of PMAA/GO hydrogels, the GO moieties are deposited on the polymer skeleton of the crosslinked network of PMAA. The pore walls act as spacers between the crosslinks, which helps with the extensive stretching of pore walls (as water enters the pores, it exerts hydrostatic pressure on the pore walls) and with holding large amounts of water molecules. When the PMAA/GO hydrogel was immersed in salt solution, its change in water absorption was as shown in Figure 2i. The figure shows that the water absorption of the PMAA/GO hydrogel decreased with the increase in the valence state; that is, the greater the charge of the cation, the more significant the impact on the swelling behavior of the hydrogel, which may be due to the positive charge and –COO^−^ ion binding, occupying the binding site of water molecules and causing its swelling properties to decrease. The swelling ratio dropped from 8.24 to 4.95.

**Figure 3:**
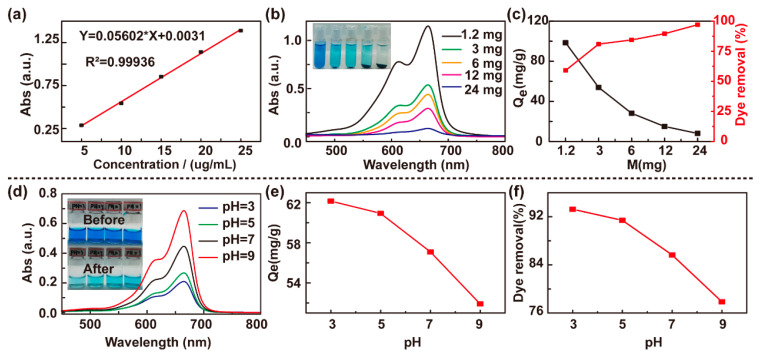
Influence of adsorbent quality and pH value on adsorption effect. (**a**) Methylene blue (MB) standard curve. (**b**) UV–vis spectra corresponding to the study of the adsorption of MB by PMAA/GO composite hydrogel of different masses. (**c**) The adsorption capacity (Qe) and dye removal of different mass. (**d**) The UV–vis spectra corresponding to the solutions of different pH values. (**e**) The relationship between the pH of the solution and the adsorption capacity. (**f**) The relationship between the pH value of the solution and the removal rate.

The standard curve of MB is shown in Figure 3a. Different masses of hybrid hydrogel PMAA/GO were immersed in 2 mL of 0.1 mg/mL MB solution and vibrated at 25 °C for 2 h. The UV–vis spectra of the MB solution (diluted two times), before and after adsorption, are shown in Figure 3b,c. As the mass of the adsorbent increased, the effect of adsorption enhanced. When the content of the adsorbent was 24 mg, the adsorption effect was the greatest. The adsorption capacity (Qe) gradually decreased and the removal ratio increased, as shown in Figure 3c, because the adsorption effect of the PMAA/GO-doped hydrogel depends on the swelling of the volume after water absorption and the hydrogen bonding, electrostatic interaction, and π–π bonding between GO molecules. With the same MB solution, the larger the mass of the adsorbent, the larger the volume after swelling, the more MB is loaded, and the larger the removal rate. The adsorption effect of the hydrogel under different pH was tested, with the results shown in Figure 3d. As the pH increased, the adsorption capacity decreased, as presented in Figure 3e. This occurred because under acidic conditions, the functional groups (such as –OH and –COOH) on the surface and the internal pore walls of the PMAA/GO hydrogel are protonated, leading to ionization, which results in an increase in the number of negative charges on the surface of the adsorbent, thereby enhancing the adsorption of the cationic dye, MB. Under alkaline conditions, the swelling rate of the hydrogel is relatively large, and the volume after swelling is also relatively large, which causes the interaction between GO molecules to restrict each other, resulting in a decrease in adsorption capacity. In addition, under alkaline conditions, the chemical reduction effect of graphene oxide is stronger than the deprotonation effect [41], which reduces the hydrophilic oxygen-containing functional groups on the surface and the number of charges. Hence, it will reduce the adsorption of positively charged MB dyes, decreasing the removal rate of MB from 93.24% (pH = 3) to 77.85% (pH = 9), as shown in Figure 3f.

**Figure 4:**
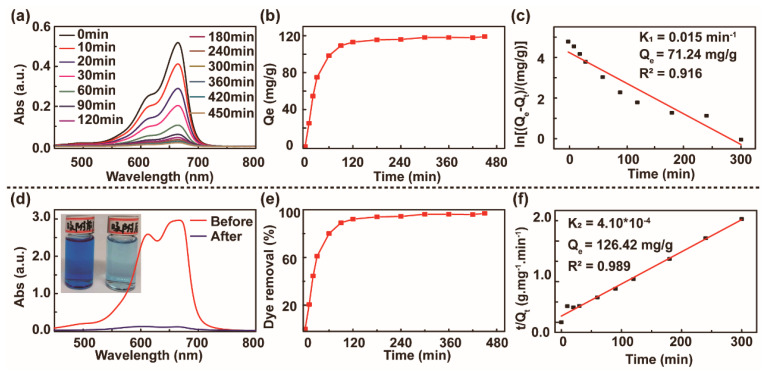
Adsorption performance studies at different times. (**a**) UV–vis spectra of the extract after different adsorption periods (all the extracts were diluted 10 times). (**b**) Diagram of the relationship between time and adsorption capacity. (**c**) Quasi-first-order dynamic model fitting curve. (**d**) UV spectrogram of the extract before and after adsorption, illustrated by comparison photos before and after adsorption. (**e**) Diagram of the relationship between time and removal rate. (**f**) Quasi-second-order dynamic model fitting curve.

The dried PMAA/GO composite hydrogel was immersed in 0.1 mg/mL MB solution. We removed a certain amount of MB solution at regular intervals until the adsorption equilibrium was reached. After being diluted 10 times, the UV–vis spectra of the MB solution removed at different times were tested, and the result is shown in Figure 4a. Figure 4b,e describe the relationship between adsorption capacity and removal rate over time. After 120 min (the adsorption capacity was 113.15 mg/g and the removal rate was 92.07%), the change slowed. After 300 min (adsorption capacity of 118.05 mg/g and removal rate of 96.14%), it gradually tended towards equilibrium, and the final adsorption capacity of MB was 119.01 mg/g, and the removal rate was 96.92%.

To further study the adsorption mechanism of the hydrogel for MB and research the kinetics of the adsorption process, we used the quasi-first-order kinetics and quasi-second-order kinetics to fit the MB adsorption data. The quasi-first-order kinetic model assumes that the occupancy rate of adsorption sites is proportional to the number of unoccupied sites. The quasi-second-order kinetic model is based on the assumption that the adsorption rate is controlled by the chemical adsorption mechanism. This chemical adsorption mechanism is determined by the adsorbate and adsorbent, which involves electron sharing or electron transfer between them. The equation of the two models is as follows [42,43]. The expression of the quasi-first order kinetic adsorption model is:

(4) $$\ln(Q_{e}-Q_{t})=\ln Q_{e}-k_{1}t$$

where Q_e_ is the equilibrium adsorption capacity, Q_t_ is the amount of adsorption of the hydrogel for MB at time *t*, *k*_1_ is the kinetic reaction rate constant, and *t* is the adsorption reaction time.

The expression of the quasi-second-order kinetic adsorption model is:

(5) $$\frac{t}{Q_{t}}=\frac{1}{k_{2}Q_{e}{}^{2}}+\frac{t}{Q_{e}}$$

where *k*_2_ is the adsorption rate constant obtained from the quasi-second-order kinetic equation.

As shown in Figure 4c,f, we plotted ln (Q_e_ − Q_t_) and *t*/Q_t_ versus *t* to obtain the quasi-first-order kinetic fitting curve and the quasi-second-order kinetic fitting curve. The obtained fitting parameters are shown in Table 1. The amount of theoretical equilibrium adsorption of the quasi-second-order kinetics agrees with the amount of equilibrium adsorption (119.01 mg/g) obtained in the experiments. The correlation coefficient of the quasi-second-order kinetics is greater than that of the quasi-first-order kinetics, which indicates that quasi-second-order kinetics is more suitable to describe the kinetic behavior of the hydrogel to MB adsorption; that is, the adsorption rate of the adsorbent mainly depends on chemical adsorption.

## 4. Conclusions

In this study, GO nanomaterials were introduced based on the PMAA hydrogel system, and PMAA/GO composite hydrogels that can adsorb the cationic dye methylene blue (MB) were obtained. The composite hydrogel was characterized by SEM, FTIR, and XRD, and the results showed that a PMAA/GO composite hydrogel was successfully produced. The swelling performance of the composite hydrogel was studied under different pH values and different salt ion solutions. The results showed that its water absorption increases with increasing pH values (from 4.44 at pH 3 to 12.4 at pH 9) and decreases with the increasing valence state from 8.24 to 4.95. In addition, taking methylene blue (MB) dye as an example, the effects of adsorbent quality, pH, and adsorption time on adsorption performance were studied, and the adsorption kinetics were analyzed. The results showed that the adsorption capacity for cationic dye MB can reach 119.01 mg/g, and the removal rate can reach 96.92%. Therefore, this work contributes to the development of new cationic adsorbents with dye removal performance, and has potential applications in the field of water treatment.

## Figures and Tables

**Table 1:** The adsorption kinetic parameters of PMAA/GO composite hydrogel for MB. R^2^: coefficient of determination; Q_e_: the equilibrium adsorption capacity; *k*_1_: the kinetic reaction rate constant; *k*_2_: the adsorption rate constant obtained from the quasi-second-order kinetic equation.

Kinetic Equations	Adsorption Kinetic Constant		R^2^
Quasi-first-order dynamics	Q_e_ = 71.24	k_1_ = 0.015	0.916
Quasi-second-order dynamics	Q_e_ = 126.42	k_2_ = 4.10 × 10^−4^	0.989

## Data Availability

Data are available on request; please contact authors using their e-mail addresses.
